# Protein–Linker Co-engineering for Broad-Spectrum Antiviral Development against Enveloped Viruses

**DOI:** 10.1021/acsmaterialslett.5c01444

**Published:** 2026-02-03

**Authors:** Lixia Wei, Colleen N. Loynachan, Gregory Mathez, Yong Zhu, Suiyang Liao, Arnaud Charles-Antoine Zwygart, Laure Menin, Caroline Tapparel, Valeria Cagno, Francesco Stellacci

**Affiliations:** † Institute of Materials Science and Engineering, 27218 École polytechnique fédérale de Lausanne, Lausanne 1015, Switzerland; ‡ Institute of Bioengineering, 27218 École polytechnique fédérale de Lausanne, Lausanne 1015, Switzerland; § Institute of Microbiology, 30635 Lausanne University Hospital, University of Lausanne, Lausanne 1011, Switzerland; ∥ Department of Microbiology and Molecular Medicine, 28535 University of Geneva, Geneva 1206, Switzerland; ⊥ Institute of Chemical Sciences and Engineering, 27218 École polytechnique fédérale de Lausanne, Lausanne 1015, Switzerland

## Abstract

Emerging and re-emerging viruses with pandemic potential pose a continuous global health threat. Broad-spectrum antivirals, if available, could serve as a critical first line of defense. Here, we present a general and simple strategy to chemically functionalize natural proteins into broad-spectrum, nontoxic antivirals. Through a one-step conjugation, proteins are modified with alkyl ligands terminated by secondary amines. These functionalized proteins exhibit potent inhibitory activity against enveloped viruses HSV-2, Influenza A H1N1, and SARS-CoV-2, with half-effective concentrations (EC_50_) ranging from nanomolar to micromolar levels. Efficacy improves with increased ligand density, and longer alkyl chains induce a shift from reversible (virustatic) to irreversible (virucidal) antiviral activity. Importantly, antiviral performance remains robust in complex serum environments, and the antiviral is most effective when administered prophylactically. This versatile platform is compatible with diverse protein scaffolds, offering a promising approach for rapid antiviral development against current and future viral threats.

## Graphical abstract

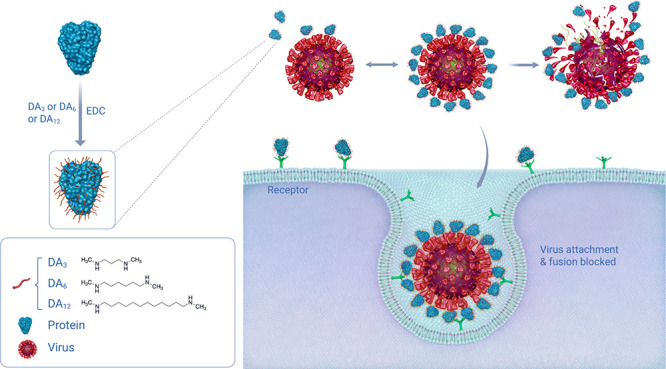

Over the past several centuries, global pandemics caused by infectious diseases have periodically threatened millions of lives and placed a heavy burden on healthcare systems worldwide. To address future threats, rapid vaccine development is a key strategy, however, this process often take years.^1^ Antiviral drugs, especially broad-spectrum ones, are highly desirable to reduce or slow infection spread, buying critical time for vaccine development.^2^ Antiviral drugs are designed to target and inhibit one or multiple stages of the viral infection cycle.^3–5^ Currently, most small-molecule antivirals target viral proteins.^6–8^ These drugs can potentially be broad-spectrum, but yet challenging.^9^ Natural proteins such as neutralizing antibodies and immune modulators (INF-) show effective inhibition against various viruses.^10–13^ However, antibodies are highly specific to individual viral protein, resulting in narrow-spectrum activity and a low barrier to resistance, while immune modulators are often associated with unwanted side effects.

A potent broad-spectrum antiviral strategy aims to block the viral attachment to the host cell membrane.^14–17^ Macromolecules including nanoparticle-,^18–22^ peptide-,^23–25^ and polymer-based antivirals^26–34^ have attracted interest as they can be engineered to multivalently display active groups that bind diverse viruses and prevent host-cell attachment. For example, materials functionalized with moieties mimicking heparan sulfate proteoglycans (HSPGs), a common cell surface receptor shared by various viruses, have been widely explored.^35^ Alternatively, cationic materials can electrostatically bind anionic viral components or interact with negatively charged HSPGs on host cells, thereby disrupting virus–cell interactions. This approach is inherently broad-spectrum, provided toxicity is controlled, and has shown efficacy for amine- and guanidine-functionalized antiviral polymers and peptides.^25^ Yet, to the best of our knowledge, most of these antivirals have a virustatic mechanism, exhibiting reversible virus binding that dissociates upon dilution and releases infectious virions. Ideal antivirals would act via a virucidal mechanism, irreversibly damaging virions and preventing infection after dilution. Our group previously developed broad-spectrum virucidal gold nanoparticles functionalized with mercapto­undecane­sulfonic acid ligands, achieving nanomolar inhibition of multiple HSPG-dependent viruses.^18^ Following initial electrostatic interactions, virucidal activity was driven by multivalent hydrophobic interactions between long alkyl chains and viral proteins residues, resulting in irreversible viral deformation and inactivation. Nevertheless, scalability and biocompatibility concerns limit the translational potential of inorganic nanoparticle-based antivirals.

Proteins are attractive scaffolds for biomaterial design due to their natural abundance, structural diversity, and excellent biocompatibility. Their surface functionalities enable precise chemical modifications and incorporation of antiviral moieties without compromising structural integrity. Compared with synthetic polymers or inorganic nanoparticles, protein-based platforms typically exhibit reduced immunogenicity and improved biodegradability, supporting systemic or mucosal administration. Here, we hypothesized that modifying a protein core with suitably hydrophobic cationic ligands would yield an effective broad-spectrum antiviral material with a tunable virucidal mechanism. Ligands were selected based on hydrophobicity and ligand density, key parameters governing virus–protein interactions by mimicking the amphiphilic nature of viral envelopes and enhancing multivalent binding. Our goal was to identify ligand characteristics that maximize viral inhibition and virucidal activity while maintaining cell compatibility, a critical requirement for therapeutic and prophylactic applications.

Here, we present a versatile approach for broad-spectrum virucidal protein-based antivirals. Bovine serum albumin (BSA), avidin, and cytochrome c (Cyto C), selected for their diverse functions, sizes, and isoelectric points, were conjugated with alkyl secondary diamine ligands. The resulting materials are termed P-DA_X_, where P denotes the protein, DA the ligand, and X the alkyl chain length. Secondary diamines were chosen for efficient conjugation, strong interactions with viral proteins, and low host-cell toxicity. Results show increasing ligand density on BSA reduced half-effective concentration (EC_50_) values, while increasing alkyl chain length had minimal effect on EC_50_ but induced a shift from reversible virustatic to irreversible virucidal activity. Importantly, antiviral efficacy was largely retained under complex serum conditions, supporting translational relevance. The platform inhibited HSV-2, Influenza A (H1N1), and SARS-CoV-2, demonstrating broad-spectrum activity with nanomolar to micromolar EC_50_ values. In vitro, maximal efficacy was observed upon prophylactic cell pretreatment, likely through binding to cell-surface HSPGs and blockade of viral entry. Overall, this protein-based platform offers a scalable, nontoxic approach for broad-spectrum antiviral intervention.

To functionalize the proteins, surface-accessible carboxylic acids (−COOH) were reacted with secondary amines (−NH−) on the ligands via carbodiimide coupling in MES buffer. Three ligands were used: N,N′-Dimethyl-1,3-propanediamine (DA_3_), N,N′-Dimethyl-1,6-hexanediamine (DA_6_), and N,N′-Dimethyldodecane-1,12-diamine (DA_12_). Protein concentrations were maintained at ≤1 mg mL^–1^ to prevent intermolecular cross-linking by the diamine ligands. The modified proteins were characterized for size (dynamic light scattering, DLS), surface charge (surface zeta potential), degree of functionalization (matrix-assisted laser desorption/ionization time-of-flight mass spectrometry, MALDI-TOF), and secondary structure (circular dichroism, CD).

Conjugation conditions were optimized using BSA and DA_3_ as exemplar protein and ligand (Table 1, entry 2; Figure 1A). BSA-DA_3_ showed an average molecular weight (MW) increase of 5711 Da, corresponding to ∼56 DA_3_ ligands per protein and ∼60% occupancy of surface-accessible acidic residues (Figure 1B). Surface modification increased the hydrodynamic diameter from 6.1 ± 1.5 nm to 8.2 ± 2.0 nm (Figure 1C) and shifted the zeta potential from −7.3 ± 3.8 mV to +31.2 ± 4.5 mV (Figure 1D). Circular dichroism confirmed that α-helix and β-sheet content were preserved after modification (Figure 1E). Analytical ultracentrifugation (AUC) revealed monomeric and dimeric species for both native and modified BSA, with shifted but similarly shaped peaks following conjugation (Figure 1F), indicating uniform surface functionalization without cross-linking or aggregation. The leftward shift in sedimentation coefficients is consistent with reduced particle density after ligand conjugation.

**Table 1:** Physicochemical Properties of Unmodified and Surface-Modified Protein-Based Antivirals

Entry	Sample	DLS (hydrodynamic diameter, nm)	Zeta potential (mV)	Mass Spectrum (MW, Da)	Number of ligands per protein
1	BSA	6.1 ± 1.5	–7.3 ± 3.8	66346	
2	BSA-DA_3_	8.2 ± 2.0	31.2 ± 4.5	72057	56
3	BSA-20%DA_3_	7.6 ± 2.0	4.7 ± 2.8	68593	22
4	BSA-10%DA_3_	7.9 ± 2.0	–0.05 ± 6.0	67713	13
5	BSA-DA_6_	7.4 ± 2.0	23.4 ± 6.0	71682	37
6	BSA-DA_12_	7.7 ± 2.0	6.4 ± 14.6	71379	22
7	Avidin	6.3 ± 1.3	5.6 ± 2.3	63564	
8	Avidin-DA_3_	8.6 ± 1.4	20.4 ± 7.2	66098	25
9	Cyto C	1.2 ± 0.3	2.0 ± 5.6	12179	
10	Cyto C-DA_3_	2.2 ± 0.3	18.2 ± 12.9	13084	9

**Figure 1:**
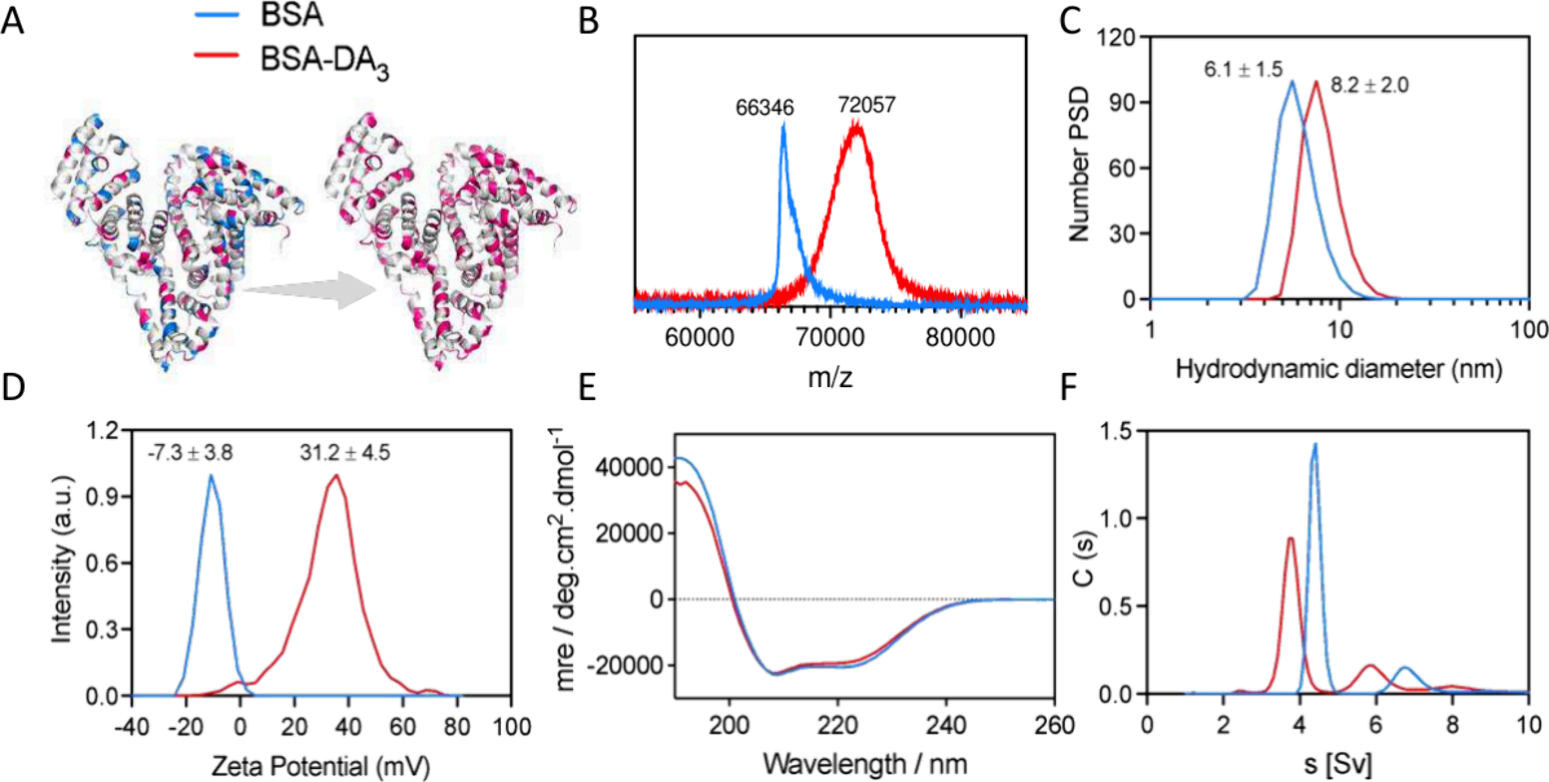
Characterization of DA_3_-modified BSA. (A) Schematic illustration of surface modification of BSA protein by ligand DA_3_, where negatively charged groups (blue) are converted into positively charged groups (red) after modification. (B) MALDI-TOF mass spectra overlay of native (blue) and modified BSA protein (red). (C) Size distribution measurement of BSA and BSA-DA_3_ by DLS. (D) Surface zeta potential of BSA and BSA-DA_3_. (E) CD spectra of native and modified BSA protein structure. (F) AUC analysis of BSA and BSA-DA_3_’s sedimentation behavior.

To define the ligand density required for antiviral activity, BSA was functionalized with DA_3_ and evaluated against HSV-2 in vitro. BSA contains 99 surface-accessible carboxylates (Asp/Glu); by varying input ligand ratios during EDC coupling (100%, 20%, 10%), we obtained BSA-DA_3_, BSA-20%DA_3_, and BSA-10%DA_3_ bearing ∼56, 22, and 13 ligands per protein, respectively. Corresponding increases in molecular weight (Figure 2A, i–iii), hydrodynamic size, and surface charge are summarized in Table 1 (entries 2–4). AUC confirmed successful functionalization across ligand densities (Figure S1), with higher ligand loading reducing particle density and resulting in slower sedimentation. Dose–response antiviral activity against HSV-2 was evaluated using a standard plaque assay (Figure 2B; Table 2). Native BSA showed no antiviral activity. In contrast, BSA-DA_3_ bearing 56 ligands exhibited potent inhibition with an EC_50_ of 3.77 μg mL^–1^ (0.052 μM) (Figure 2B, i; Table 2, entry 2). Reducing ligand density to 22 ligands resulted in a ∼400-fold loss of potency (EC_50_ = 1.62 mg mL^–1^, 23.6 μM) (Figure 2B, ii; Table 2, entry 3), while no inhibition was observed for BSA modified with 13 ligands (Figure 2B, iii; Table 2, entry 4). Thus, neither unmodified BSA nor low-density conjugates exhibited antiviral activity, highlighting the critical role of ligand density and multivalent presentation in mediating efficacy. Increased ligand density also correlated with higher surface charge, enhancing multivalent ionic interactions with viral particles and likely imposing greater stress on viral proteins during virus pretreatment. Cell viability assays confirmed that antiviral activity was not associated with host-cell toxicity. All modified BSA conjugates maintained >95% cell viability across the concentration range used in antiviral assays (Figure 2C, i–iii), demonstrating their nontoxic profile.

**Figure 2:**
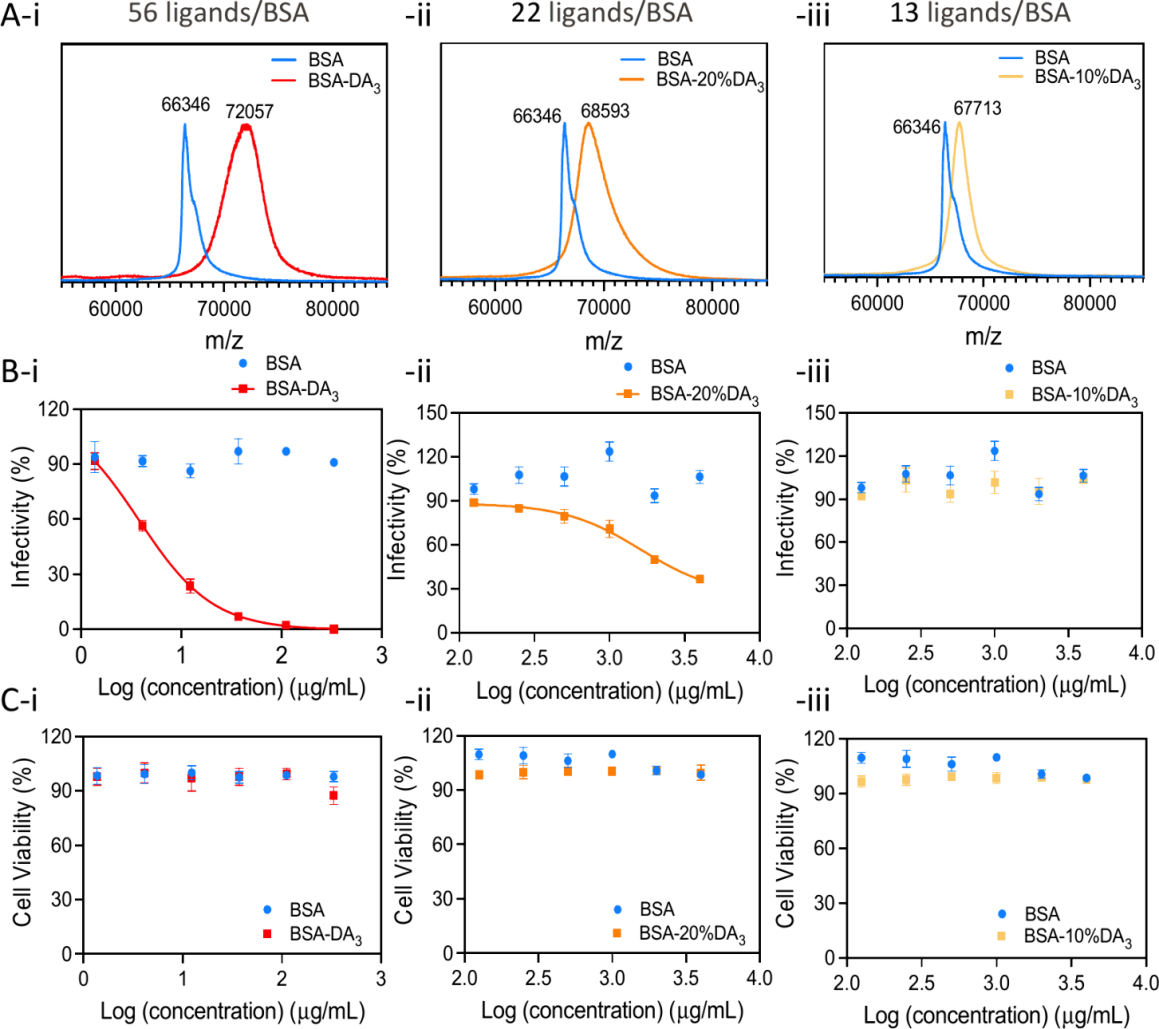
Ligand density influence on antiviral effect. (A, i–iii) Different ligand density products characterized by MALDI-TOF mass spectrometry: i, BSA-DA_3_ with 56 ligands conjugated has size around 8.2 ± 2.0 nm and surface charge around 31.2 ± 4.5 mV; ii, BSA-20%DA_3_ with 22 ligands conjugated has size around 7.6 ± 2.0 nm and surface charge around 4.7 ± 2.8 mV; iii, BSA-10%DA_3_ with 13 ligands conjugated has size around 7.9 ± 2.0 nm and surface charge around −0.05 ± 6.0 mV. (B, i–iii) Dose–response viral infectivity of different ligand density BSA-DA_3_. (C, i–iii) Cytotoxicity of different ligand density BSA-DA_3_ on host Vero cells.

**Table 2:** Protein-Based Antivirals’ Viral Inhibition Effect against HSV-2^a^

Entry	Sample	Number of ligands per protein	EC_50_ (μg/mL)	EC_50_ (μM)	Virucidal	Cell Viability (%)
1	BSA	0	NA	NA	No	>95%
2	BSA-DA_3_	56	3.77	0.052	No	>95%
3	BSA-20%DA_3_	22	1620	23.6	No	>95%
4	BSA-10%DA_3_	13	NA	NA	No	>95%
5	BSA-DA_6_	37	0.43	0.006	No	>95%
6	BSA-DA_12_	22	10.66	0.149	Yes	>95%
7	Avidin	0	NA	NA	No	>90%
8	Avidin-DA_3_	25	13.76	0.208	No	>90%
9	Cyto C	0	NA	NA	No	>90%
10	Cyto C-DA_3_	9	7.75	0.592	No	>75%

a The antiviral activity against HSV-2 reported here was tested in all cases by standard dose–response inhibition assay and standard virucidal assay as indicated in the Supporting Information.

To assess the role of ligand hydrophobicity, BSA was functionalized with alkyl diamine ligands of increasing chain length: DA_3_, DA_6_, and DA_12_ (Figure 3A). Based on the importance of ligand density, conjugation was performed under conditions maximizing ligand loading for each ligand length. BSA-DA_3_, BSA-DA_6_, and BSA-DA_12_ were obtained using identical synthetic protocols (Table 1, entries 2, 5, and 6). MALDI-TOF analysis revealed average ligand loadings of 56 (DA_3_), 37 (DA_6_), and 22 (DA_12_) per BSA, with decreasing conjugation efficiency at longer chain lengths likely due to steric hindrance. AUC confirmed homogeneous functionalization without cross-linking for all samples (Figure S2). Antiviral activity tests against HSV-2 showed EC_50_ values of 0.052 μM (BSA-DA_3_), 0.006 μM (BSA-DA_6_), and 0.149 μM (BSA-DA_12_) (Figure 3B; Table 2, entries 2, 5, and 6), with no monotonic trend, reflecting a balance between ligand hydrophobicity and ligand density. Notably, when ligand density was matched (22 ligands per protein), BSA-DA_12_ (0.149 μM) was ∼158-fold more potent than BSA-20%DA_3_ (23.6 μM), demonstrating the positive contribution of increased hydrophobicity. All conjugates maintained >95% cell viability over the concentration range tested (Figure S3). To further assess the role of ligand hydrophobicity in the antiviral mechanism, virucidal assays were performed. BSA-DA_3_, BSA-DA_6_, and BSA-DA_12_ at EC_99_ concentrations were incubated with HSV-2 (10^6^ pfu), followed by serial dilution and plaque assays. It revealed a clear dependence on ligand hydrophobicity as BSA-DA_3_ and BSA-DA_6_ showed reversible inhibition, whereas BSA-DA_12_ reduced viral titers by ∼10^3^-fold after dilution, indicating irreversible virucidal activity (Figure 3D). These results demonstrate that while both ligand density and hydrophobicity contribute to antiviral potency, virucidal activity is primarily governed by hydrophobicity. The longer alkyl chain of DA_12_ likely enables sufficient physical disruption of viral envelopes to induce irreversible virion inactivation, consistent with prior observations that a hydrophobicity threshold is required for virion deformation.

**Figure 3:**
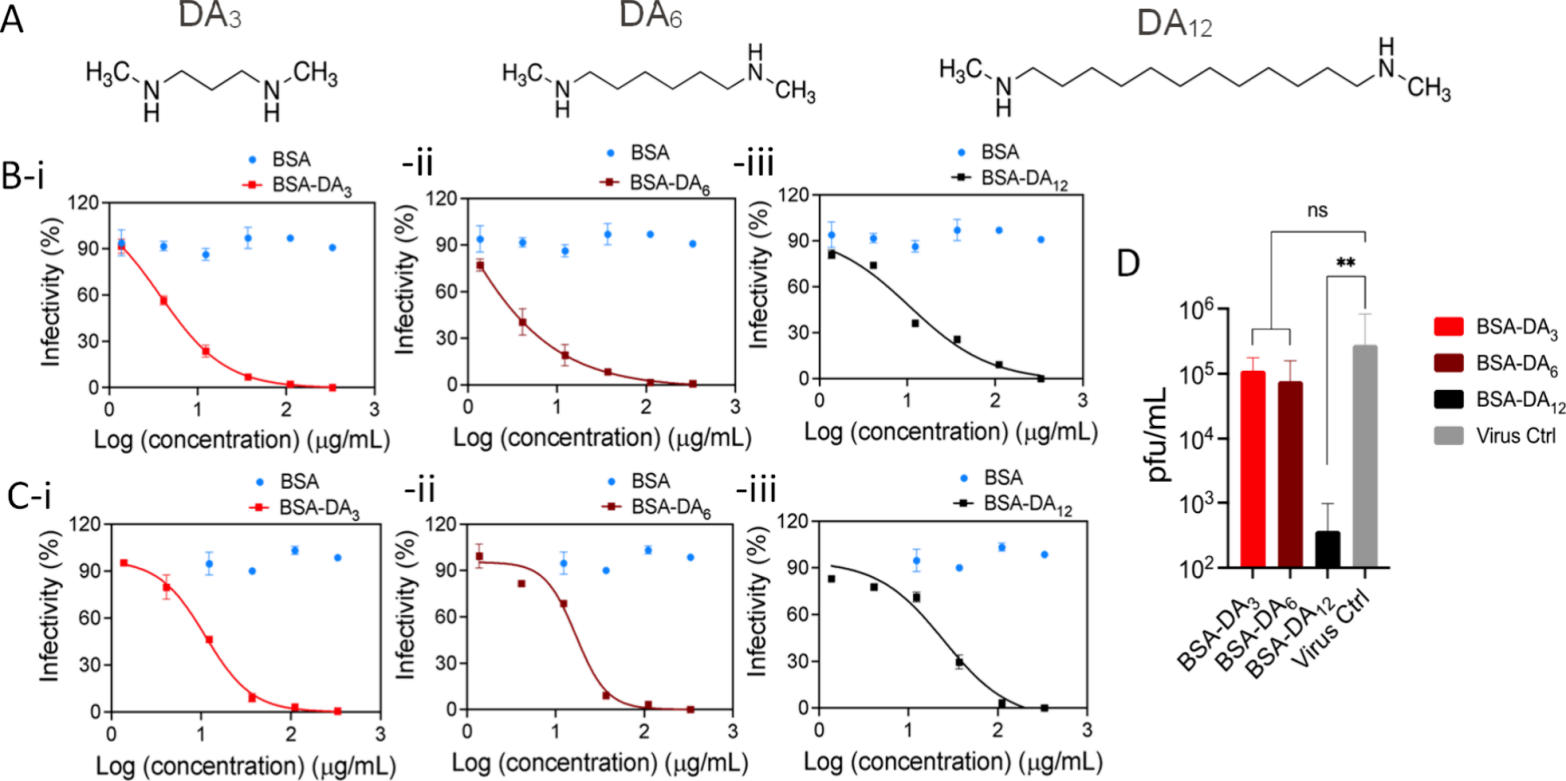
Effect of ligand hydrophobicity on antiviral inhibition against HSV-2 by varying alkyl chain length (DA_3_, DA_6_, and DA_12_). (A) Chemical structures of varying ligand hydrophobicity DA_3_, DA_6_, and DA_12_. (B, i–iii) Dose–response viral infectivity of antivirals with varying hydrophobicity. (C, i–iii) Influence of 55% serum protein environment on antiviral inhibition. (D) Virucidal effect of these protein antiviral materials with varying hydrophobicity. Results are expressed as averages and standard errors from two independent experiments conducted in duplicate. Statistical significance was analyzed with a two-tailed unpaired *t* test. The asterisks represent the P value (*, <0.05; **, <0.01; ***, <0.001).

Stability under complex serum conditions is critical for translational potential; therefore, we investigated the effect of serum proteins on antiviral activity. Standard dose–response viral infection assays are typically performed in 2% fetal bovine serum (FBS) containing cell culture medium, whereas human blood contains ∼55% serum proteins, which can reduce antiviral efficacy through nonspecific binding.^36,37^ To assess serum effects, the conjugates were preincubated in 100% FBS to achieve a final serum concentration of 55% for 1 h at 25 °C with stirring (600 rpm), followed by standard dose–response viral infection assays. As shown in Figure 3C, serum exposure resulted in variable EC_50_ shifts: BSA-DA_3_ increased ∼3-fold (0.052 to 0.158 μM), BSA-DA_6_ increased ∼41-fold (0.006 to 0.246 μM), and BSA-DA_12_ increased ∼2.6-fold (0.149 to 0.392 μM). While BSA-DA_6_ exhibited a larger rightward shift, its absolute potency remained in the low micromolar range (<0.25 μM), indicating retained antiviral activity under high-serum conditions. Overall, these results demonstrate that this protein-based antiviral strategy maintains functional efficacy in the presence of excess serum proteins, supporting its translational relevance.

Given high viral mutation rates, broad-spectrum antivirals are highly desirable. To assess antiviral breadth, we evaluated the protein-based platform against three enveloped viruses: HSV-2, Influenza A H1N1, and SARS-CoV-2 (Figure 4A). BSA-DA_12_ was selected as a representative antiviral due to its virucidal effect against HSV-2. BSA-DA_12_ was selected as a representative compound due to its virucidal activity against HSV-2. Dose–response assays yielded EC_50_ values of 0.149 μM for HSV-2 (Figure 4B, i), 4.48 nM for Influenza H1N1 (Figure 4B, ii), and 2.42 μM for the SARS-CoV-2 alpha variant (Figure 4B, iii). Cytotoxicity assays across the same concentration ranges in Vero, MDCK, and Vero E6 cells showed >90% viability (Figure 4C, i–iii), confirming good biocompatibility. Virucidal assays indicated that BSA-DA_12_ was virucidal against HSV-2 but not against Influenza H1N1 or SARS-CoV-2 (Figure 4D), likely reflecting differences in viral surface properties and the binding or hydrophobic forces required for irreversible damage. This study focuses on enveloped viruses with lipid membranes susceptible to surface disruption; therefore, while broad-spectrum inhibition was observed, virucidal activity remains virus-dependent. Extension of this strategy to non-enveloped viruses will require further investigation and potentially alternative functionalization approaches.

**Figure 4:**
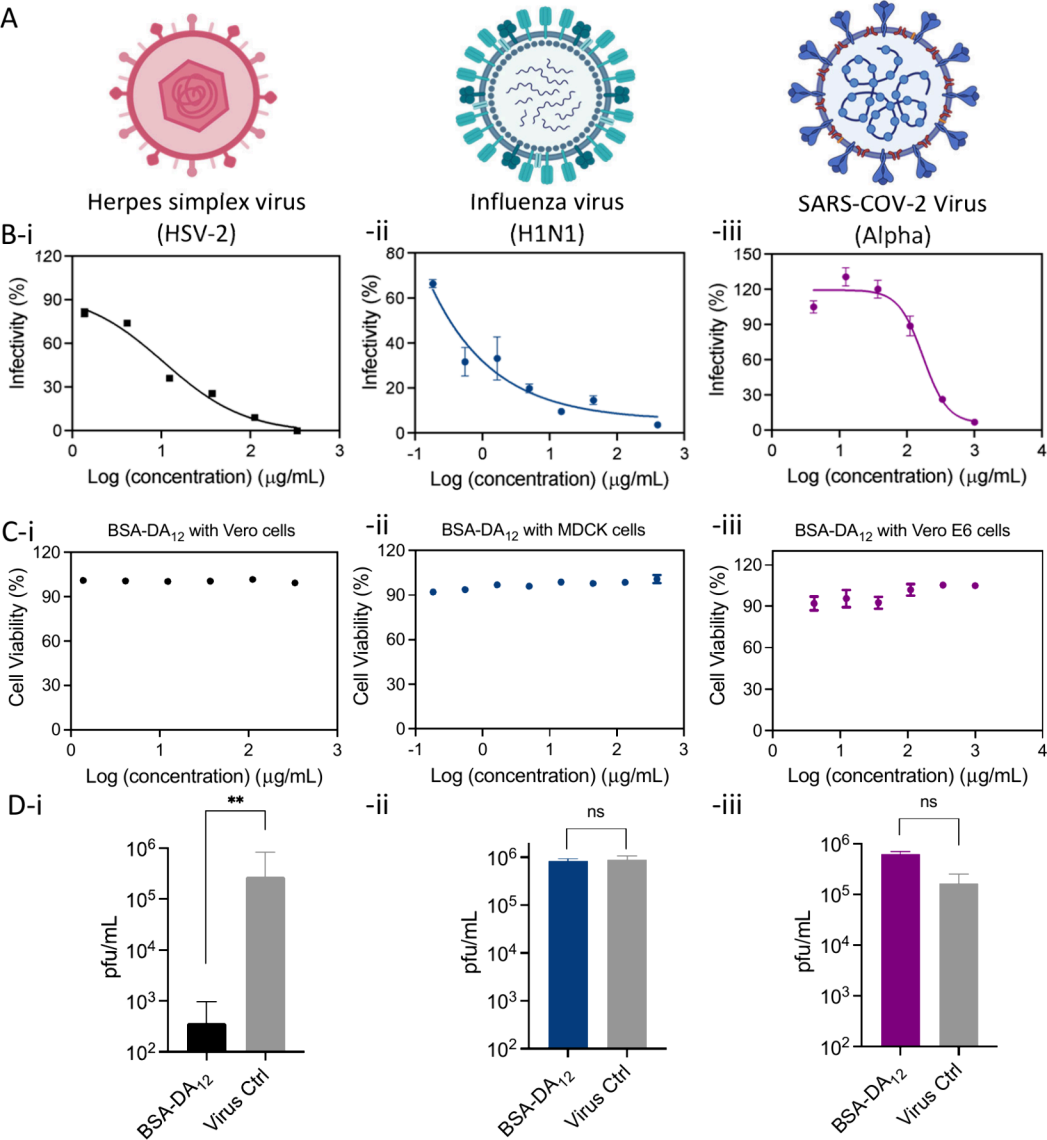
Broad-spectrum antiviral inhibition of BSA-DA_12_ against HSV-2, Influenza H1N1, and SARS-CoV-2 viruses. (A) Schematic structure of three different types of viruses: HSV-2, Influenza H1N1, and SARS-CoV-2. (B) Dose–response viral infectivity of BSA-DA_12_ against HSV-2 (-i), Influenza H1N1 (-ii), and SARS-CoV-2 (-iii). (C) Cell viability of antivirals BSA-DA_12_ on host cells Vero cell (-i), MDCK cell (-ii), and Vero E6 cell (-iii). (D) Virucidal effect of antivirals BSA-DA_12_ against viruses HSV-2 (-i), Influenza H1N1 (-ii), and SARS-CoV-2 (-iii). Results are expressed as averages and standard errors from two independent experiments conducted in duplicate. Statistical significance was analyzed with a two-tailed unpaired *t* test. The asterisks represent the P value (*, <0.05; **, <0.01; ***, <0.001).

We further evaluated different treatments of these protein-based materials on antiviral effect: (1) virus pre-treatment, with viruses and antiviral materials preincubated for 1 h at 37 °C before infection; (2) co-treatment, with viruses and antiviral materials added simultaneously without pre-incubation; (3) post-treatment, with antiviral materials added 1 h after viral infection; and (4) cell pre-treatment, with cells pre-incubated with antiviral materials for 1 h at 37 °C prior to infection. All experiments used the virucidal conjugate BSA-DA_12_ against HSV-2. As shown in Figure S4A-D, virus pre-treatment with BSA-DA_12_ yielded an EC_50_ of 0.149 μM (Figure S4A). Co-treatment resulted in a comparable EC_50_ of 0.186 μM (Figure S4B), indicating similar efficacy without pre-incubation. Under post-treatment (therapeutic) conditions, the EC_50_ increased to 0.341 μM (Figure S4C), consistent with a mechanism that inhibits viral attachment rather than post-entry events. In contrast, cell pre-treatment produced the greatest potency, with an EC_50_ of 0.028 μM (Figure S4D), indicating that pre-conditioning host cells substantially enhances antiviral efficacy.

Macromolecular antivirals that block viral attachment often show limited efficacy under cell pre-treatment conditions.^18,38^ To understand the enhanced activity of BSA-DA_12_, we examined its association with host cells. Alexa-647 dye-labeled native BSA and BSA-DA_12_ were incubated with Vero cells for 1 h at 37 or 4 °C and analyzed by flow cytometry. Native BSA showed negligible cell association, whereas BSA-DA_12_ exhibited >90% association at 37 °C and >30% at 4 °C (Figure S4E). Confocal imaging confirmed strong surface adhesion of BSA-DA_12_ at both temperatures, in contrast to native BSA (Figure S4F), with no evidence of significant internalization. This is consistent with electrostatic binding of positively charged BSA-DA_12_ to negatively charged cell-surface HSPGs, effectively masking viral attachment sites and preventing HSV-2 entry.

To assess platform versatility, the same DA_3_ modification strategy was applied to proteins with distinct molecular weights and isoelectric points: BSA (67 kDa, pI 4.7), avidin (67 kDa, pI 10), and cytochrome c (Cyto C; 11.7 kDa, pI 9.6) (Figure 5A). Characterization of size, surface charge, and ligand loading is summarized in Table 1 (entries 2, 8, and 10) and Figure S5. The ligand conjugation efficiency is primarily determined by the number of surface-accessible binding sites (carboxyl groups), estimating respectively BSA 90/99, Avidin 40/44, and Cyto C 11/12. Despite differences in protein size and native surface charge, all modified proteins exhibited effective HSV-2 inhibition with EC_50_ values <1 μM: BSA-DA_3_ (56 ligands, 0.052 μM), avidin-DA_3_ (25 ligands, 0.208 μM), and Cyto C-DA_3_ (9 ligands, 0.592 μM) (Figure 5B; Table 2, entries 2, 8, 10). Cytotoxicity assays showed >90% cell viability for native proteins, BSA-DA_3_, and avidin-DA_3_ across tested concentrations, while Cyto C-DA_3_ displayed reduced viability only at the highest dose (333 μg mL^–1^), consistent with the known pro-apoptotic activity of cytosolic cytochrome c (Figure S6, Table 2, entries 1, 2, 7, 8, 9, 10). These results demonstrate the modularity of this protein-based antiviral platform and its applicability to diverse protein cores, including antibodies or enzymes. Nevertheless, preservation of functional domains and conformational stability must be carefully evaluated for structurally or functionally sensitive proteins prior to conjugation.

**Figure 5:**
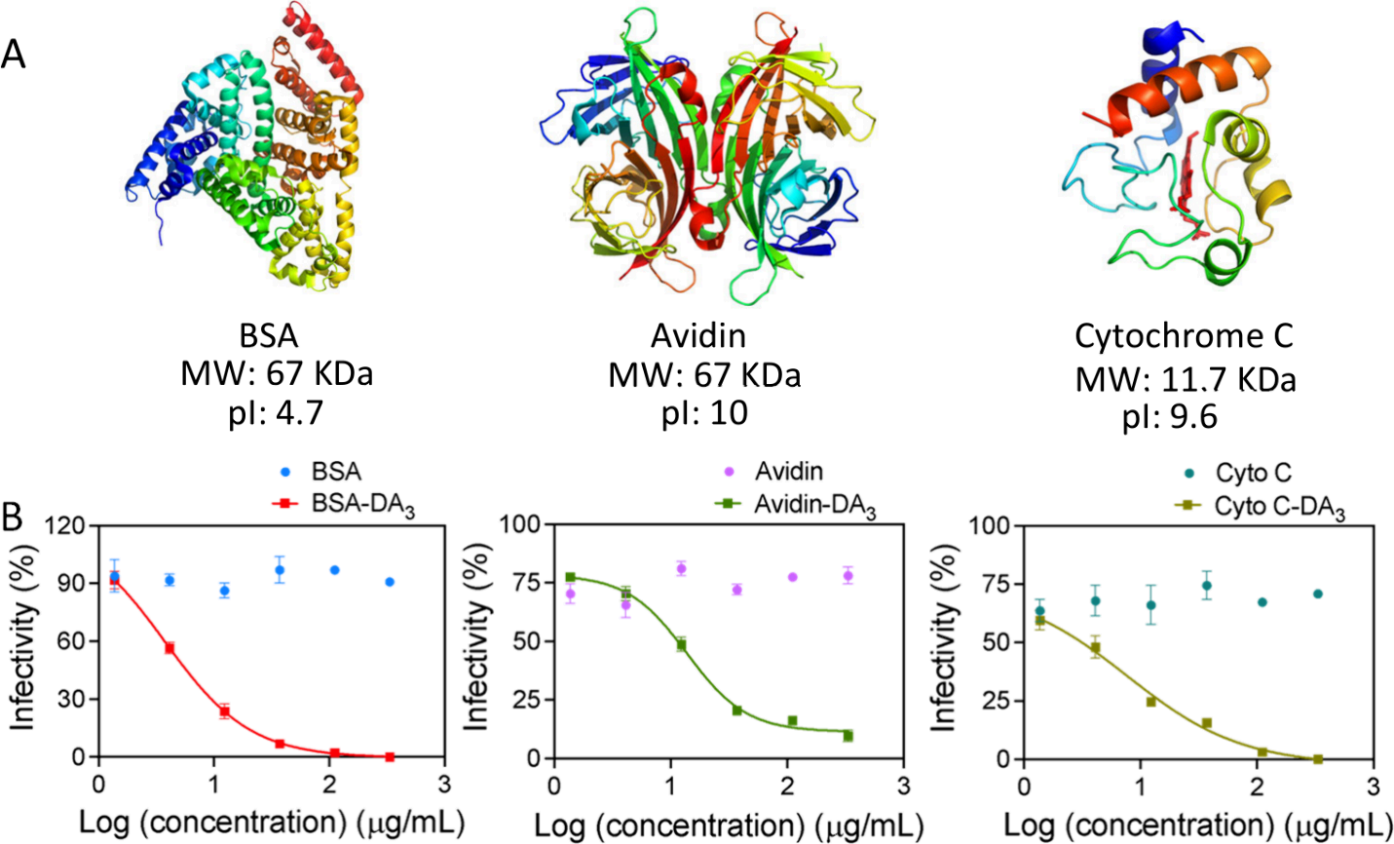
Different protein core-based antiviral materials’ influence on antiviral inhibition effect against HSV-2. (A) Schematic structure of different proteins (BSA, Avidin, and cytochrome C) with their molecular weight and isoelectric point. (B) Dose–responsive viral infectivity of different protein core-based materials’ treatment. Assay was performed against HSV-2 with standard virus pre-treatment for 1 h at 37 °C, where each modified protein was tested with concentrations of 1.4 μg/mL, 4 μg/mL, 12 μg/mL, 37 μg/mL, 111 μg/mL, and 333 μg/mL. (The images shown for the BSA-DA_3_ group in Figures 2B-i, 3B-i, and 5B are intentionally duplicated, as they originate from the same experimental data set. They are repeated across figures to enable direct comparison with other treatment groups under consistent conditions.)

Overall, our protein–ligand conjugates differ fundamentally from direct-acting antivirals (DAAs), which target specific viral enzymes and are vulnerable to resistance from viral mutation. By mimicking viral surface features and physically disrupting viral entry, this platform offers a broader, mutation-resilient mechanism. Unlike monoclonal antibodies that require precise antigen recognition, our approach relies on nonspecific physicochemical interactions, enabling versatile activity against diverse enveloped viruses. Compared with nanoparticle- or peptide-based entry inhibitors, the use of naturally occurring proteins conjugated with hydrophobic ligands provides intrinsic biocompatibility, low immunogenicity, and scalable production. The robust antiviral and virucidal activity observed under high-serum conditions further supports relevance in complex biological environments.

Besides, the potential for off-target effects on cellular transport was evaluated by examining the penetration mechanism of our co-engineered proteins.^39^ Mechanistic studies using endocytic inhibitors and temperature-controlled assays indicate that these molecules primarily utilize a direct cytosolic entry pathway. This bypasses the typical endosomal–lysosomal route, suggesting that the modified proteins do not significantly sequester the endocytic machinery required for nutrient and signaling molecule uptake. Furthermore, the cytosolic localization ensures that the antiviral proteins are eventually cleared by the cell’s endogenous proteasomal degradation pathways, avoiding long-term intracellular accumulation and associated toxicity. Given the broad-spectrum efficacy and modular design of our materials, this strategy could be applied in both prophylactic and therapeutic settings, including surface disinfectants, nasal sprays, or even systemic formulations pending future pharmacokinetic and toxicological evaluations.

In summary, we have demonstrated protein-based antivirals as an easily manufactured, nontoxic, and versatile platform for broad-spectrum antiviral efficacy. Through a one-step simple chemical functionalization approach at room temperature, we generated reproducible protein conjugates that exhibit potent antiviral inhibition against HSV-2, Influenza A H1N1, and SARS-CoV-2, as well as virucidal activity against HSV-2. Ligand density and hydrophobicity emerged as key parameters governing antiviral potency and virucidal behavior. Built on naturally biocompatible protein cores, either inert or functional, this platform retains efficacy under serum-rich conditions, supporting its potential use in prophylactic settings. The observed broad-spectrum activity highlights the value of modular antiviral platforms for responding to emerging and re-emerging viral threats.

Future work will focus on tuning chemical functionalities to enhance selectivity, mimicking viral envelope charge and hydrophobic domains to strengthen binding and inhibition, and validating in vivo performance, including biodistribution, pharmacokinetics, immunogenicity, and therapeutic efficacy. This initial demonstration using readily available protein cores establishes a scalable and cost-effective strategy for pandemic preparedness. Unlike vaccines or antibody therapies that require long development timelines and cold-chain logistics, this platform enables rapid customization and deployment, including in low-resource settings, offering a promising countermeasure against viral evolution, zoonotic spillovers, and future global health emergencies.

## Supplementary Material



## Notes

Materials used in experiments, including cell lines, viruses, and instruments; experimental methods including synthesis, characterizations, antiviral tests, cell penetration, confocal imaging, and cytotoxicity tests; supplemental figures (PDF)
