# Supplementary material for: Protein–Linker Co-engineering for Broad-Spectrum Antiviral Development against Enveloped Viruses
Source: ACS Mater Lett. 2026 Feb 3;8(3):788–96. doi: 10.1021/acsmaterialslett.5c01444 (PMC12958346; doi:10.1021/acsmaterialslett.5c01444)
Supplement: Supplementary file 1 [file tz5c01444_si_001.pdf]

Supporting Information

Protein-linker co-engineering for broad-spectrum antiviral development against enveloped viruses

Lixia Wei[1,2], Colleen N. Loynachan[1], Gregory Mathez[3], Yong Zhu[1,2], Suiyang Liao[1,2], Arnaud Charles-Antoine Zwygart[4], Laure Menin[5], Caroline Tapparel[4], Valeria Cagno[3], Francesco Stellacci*[1,2]

[1]Institute of Materials Science and Engineering, École polytechnique fédérale de Lausanne, Lausanne, 1015, Switzerland

[2]Institute of Bioengineering, École polytechnique fédérale de Lausanne, Lausanne, 1015, Switzerland

[3]Institute of Microbiology, Lausanne University Hospital, University of Lausanne, Lausanne, 1011, Switzerland

[4]Department of Microbiology and Molecular Medicine, University of Geneva, Geneva, 1206, Switzerland

[5]Institute of Chemical Sciences and Engineering, École polytechnique fédérale de Lausanne, Lausanne, 1015, Switzerland

*Email: francesco.stellacci@epfl.ch

Materials

Protein BSA and Cytochrome C were purchased from Sigma Aldrich (Missouri, United States). Protein Avidin is from Santa Cruz Biotechnology (Texas, United States). Chemicals 1-Ethyl-3-(3-dimethylaminopropyl) carbodiimide hydrochloride (EDC), N, N′-Dimethyl-1,3-propanediamine (DA_3), N, N′-Dimethyl-1,6-hexanediamine (DA_6), N, N'-Dimethyldodecane-1,12-diamine (DA_{12}), Avicel, 4% PFA solution, Tween 20, and 3,3'-diaminobenzidine (DAB) tablet were purchased from Sigma Aldrich (Missouri, United States). Methylcellulose was purchased from Fluka (St. Gallen, Switzerland), and Crystal violet is from Acros (Geel, Belgium). Primary antibody (Influenza A antibody) was purchased from Light Diagnostics (Missouri, United States). Secondary antibodies (Anti-mouse IgG, HRP-linked antibody) were purchased from Cell Signaling Technology (Massachusetts, United States). Alexa Fluor™ 647 N-hydroxysuccinimide (NHS) ester, DAPI solution, ProLong™ diamond antifade mountant, and Hoechst 33342 were purchased from BioLegend (San Diego, California, United States). Cell culture related materials such as medium DMEM, fetal bovine serum (FBS), Penicillin-Streptomycin (P/S) (10,000 U/mL), Trypsin-EDTA (0.25%), PBS pH 7.4 (1X) were purchased from Life Technology (California, United States). The MTS cytotoxicity assay kit was purchased from Promega (Wisconsin, United States). Unless otherwise noted, all chemical and biological reagents were used as received. All solvents purchased were reagent grade.

Cell line

Vero cells (African green monkey fibroblastoid kidney cells) were purchased from ATCC (CCL-81) and cultured in DMEM, high glucose, GlutaMAX Supplement, pyruvate supplemented by fetal bovine serum (FBS, 10%), Penicillin (100 UmL^{-1}), Streptomycin (100 μgmL^{-1}). Cells were cultured in a humidified atmosphere with 5% of CO_2 at 37 °C.

MDCK cells (Madin-Darby canine kidney cells) were purchased from ATCC (American Type Culture Collection, Rockville, MD) and cultured in DMEM, high glucose, GlutaMAX Supplement, pyruvate supplemented by fetal bovine serum (FBS, 10%), Penicillin (100 UmL^{-1}), Streptomycin (100 μgmL^{-1}). Cells were cultured in a humidified atmosphere with 5% of CO_2 at 37 °C.

Vero C1008 (clone E6) cells (ATCC, CCL-1586) were a kind gift from Prof. Gary Kobinger, and cultured in DMEM, high glucose, GlutaMAX Supplement, pyruvate supplemented by fetal bovine serum (FBS, 10%), Penicillin (100 UmL^{-1}), Streptomycin (100 μgmL^{-1}). Cells were cultured in a humidified atmosphere with 5% of CO_2 at 37 °C.

Virus

Herpes Simplex Virus type 2 (HSV-2) was clinically isolated and provided by Prof. M. Pistello (University of Pisa, Italy). HSV-2 strains were propagated and titrated by plaque assay on Vero cells.

Influenza H1N1 Neth09 was clinically isolated and provided by Prof. M. Schmolke (University of Geneva). These strains were propagated and titrated by Immunocytochemistry (ICC) on MDCK cells.

SARS-CoV-2 B.1.1.7 (hCoV-19/Switzerland/VD-CHUV-GEN3159/2021) was isolated from a clinical specimen from the University Hospital of Lausanne (CHUV) as described.[1] These strains were propagated and titrated by plaque assay on Vero-E6 cells.

Instruments

Matrix-assisted laser desorption/ionization time-of-flight mass spectra (MALDI-TOF-MS) were acquired on an Autoflex Speed (Bruker, Billerica, Massachusetts, USA). Protein-based antivirals' size and surface zeta potential were characterized by dynamic light scattering (DLS) and Zeta Potential on Malvern NanoZS (Worcester, UK). MTS absorbance was measured with a microplate reader Tecan Infinite 200Pro (Männedorf, Switzerland). Protein structure integrity was measured with a circular dichroism spectrometer (CD) Chirascan V100 from Applied Photophysics (Leatherhead, UK). Analytical Ultracentrifugation (AUC) Beckman

Optima XL-A, An-60 Ti rotor (California, United States). Cell penetration data were acquired using an Attune NxT flow cytometer (Thermo Fisher Scientific). Confocal fluorescent microscope images were acquired with an Inverted Leica DMi8 with 40X oil objectives (Leica, Wetzlar, Germany).

Methods

Preparation of protein-based antivirals

To prepare these protein-based antivirals, three solutions were prepared separately first. Solution 1: proteins BSA or Avidin or Cytochrome C were dissolved in MES pH = 4.7 buffer (30 mg into 7.5 mL buffer with concentration 4 mg/mL) Solution 2: ligands such as DA_3 or DA_6 or DA_{12} were weighted 600 mg, and dissolved in 1-2 mL miliq water, adjust pH to ~7.0 by adding 1M HCl, top-up the final volume into 15 mL with miliq water, final ligand concentration is 40 mg/mL. Solution 3: EDC 300 mg was dissolved in 7.5 mL MES pH = 4.7 buffer, the final concentration of EDC solution is 40 mg/mL. In this reaction, the mass ratio of protein, ligand, and EDC is fixed into Protein : Ligand : EDC = 1 : 20 : 10. Mix solutions 1, 2, 3 together with magnetic stirring at around 600 rpm overnight under room temperature. Purification of the final products was carried out by using an Amicon filter tube with molecular cutoff 30 KDa for BSA and Avidin protein and 10 KDa for Cytochrome C protein, miliq water was top-up to around 10 mL for washing at least 5 times with centrifugation under 5000 rpm speed for 5 mins.

Sample BSA-20%DA_3 and BSA-10%DA_3 synthesis keep the same amount of protein (Solution 1), only decrease the input ligand density to above 20% (solution 2: ligand 8 mg/mL, 15 mL, pH adjusted into 7.0) and 10% (solution 2: ligand 4 mg/mL, 15 mL, pH adjusted into 7.0). EDC dose also decreased accordingly to above 20% (solution 3: EDC 8 mg/mL, 7.5 mL) and 10% (solution 3: EDC 4 mg/mL, 7.5 mL). Sample BSA-20%DA_3 uses the fixed mass ratio of Protein : Ligand : EDC = 1 : 4 : 2, sample BSA-10%DA_3 uses the fixed mass ratio of Protein : Ligand : EDC = 1 : 2 : 1.

Characterization of protein-based antivirals

DLS: All purified protein-based antiviral product solutions were in miliq water with a concentration of 1 mg/mL. Eppendorf disposable cuvette with an absorbance range of 220 - 1600 nm was used. 100 μL volume was put in the cuvette and measured by instrument Malvern NanoZS with condition manually scan for 10 runs under room temperature.

Zeta Potential: All purified protein-based antiviral product solutions were in miliq water with 1 mM KCl in concentration 1 mg/mL. Malvern disposable folded capillary cells DTS1070 were used for the measurement in the instrument Malvern NanoZS.

Mass Spectrum: MALDI-TOF analyses were performed on an Autoflex Speed time-of-flight mass spectrometer (Bruker Daltonics, Bremen, Germany) equipped with a Bruker smartbeamTM-II laser (355 nm wavelength) and operated in the linear positive mode. Ion source 1 was set to 19.6 kV, ion source 2 was set to 17.5 kV, Pulsed Ion Extraction was set to 28 kDa, and the mass range for detection was set up based on the specific proteins. Spectra were acquired using flexControl version 3.4. The three-layer method was used to spot the samples. Briefly, sinapinic acid (SA, Merck) matrix solution at 20 mg/mL in acetone was deposited on each spot of an MTP 384 ground steel BC target plate (Bruker) and allowed to dry again at room temperature, forming a very thin first layer of matrix. The sample was centrifuged at 10000 × g for 2 minutes and 0.5 µL of the supernatant was spotted on the target and allowed to dry at room temperature. After drying, 0.5 µL of SA matrix solution at 10 mg/mL in acetonitrile 50%, water 49.9%, and trifluoracetic acid 0.1% was applied to each spot and allowed to dry again at room temperature. Each spectrum was collected as a minimum of 2000 shots. For each measurement, the spectra were processed using flexAnalysis 3.4 Compass 1.4 (Bruker Daltonics, DE). For calibration, 0.5 L of Bruker Protein Standard II was deposited using the same method as the supernatant.

Circular dichroism (CD): CD spectroscopy was used to analyze the effect of the cationization process on protein secondary structure. BSA has a primarily alpha-helical structure, where the CD spectrum has negative bands at 222 nm and 208 nm and a positive band at 196 nm. The secondary structure of BSA does not change significantly upon cationization, exhibiting a minimal increase in beta-sheet content and a concomitant decrease in alpha-helical content. CD experiments were performed on an Applied Photophysics Chirascan V100 Spectropolarimeter with quartz cells (l = 0.1 cm). Spectra were obtained from aqueous solutions (0.1 – 0.2 mg ml 1 in 50 mM phosphate buffer), and data was collected with 1 nm steps between 260 – 180 nm and 2 second integration time per step. A minimum of three spectra was recorded for static scans at 25 °C.

Analytical Ultracentrifugation (AUC): AUC was performed using a Beckman Optima XL-A, An-60 Ti rotor. All sample solutions were prepared freshly in PBS buffer to obtain final solutions that had 0.5~1.0 OD (optical density) absorbance at 280 nm in AUC cells (double sector titanium centerpieces with quartz windows; the optical path length is 1.2 cm). All measurements were made at 20 ℃, 50,000 rpm. (with a radial step size of 0.003 cm) with sufficient duration to ensure complete sedimentation. Data ranges from 50-100 scans were chosen to represent the whole transporting process.

Standard Dose-response Inhibition Plaque Assays against HSV-2

The antiviral effect of these protein-based materials against HSV-2 infection was evaluated by a standard dose-response plaque reduction assay. Vero cells were plated in a 24-well plate around 24 hours in advance with a cell seeding density of 10^5 cells/well. Protein-based antiviral materials were prepared into 1 mg/mL concentration in miliq water. Six 1.5 mL Eppendorf tubes were prepared for each protein sample with 500 uL DMEM containing 2% FBS filled inside. Take 250 μL of 1 mg/mL sample and add into the first tube, mix well with 500 μL DMEM medium, then take another 250 μL out from the first tube and transfer into the next one, 1 : 3 serial dilutions were performed like this until reaching the last tube. In the last tube after mixing, 250 μL volume was taken out and disposed to keep every tube volume constant before incubation with viruses. HSV-2 virus titer of 40000 pfu/mL was first diluted 5 times with DMEM medium with 2% FBS into 8000 pfu/mL, then 10 μL was added into each tube containing different concentrations of materials, mixed well, and incubated at 37 °C for 1 hour. Vero cells culture medium was aspirated and added virus-material mixture after incubating each well at 200 μL and incubated at 37 °C for 1 hour. Following virus adsorption, the virus inoculum was removed, and cells were washed with a medium and then overlaid with 500 μL medium containing 1.2 % methylcellulose. After incubation at 37 °C in the cell culture incubator overnight, methylcellulose methylcellulose-containing medium was removed and cells were fixed and stained with 300 μL 0.1 % of crystal violet in 20% ethanol for 15 mins, followed by washing with PBS 7.4 (1X) twice and drying the well. Viral plaques were counted. The concentration producing a 50% reduction in plaque formation (EC_{50}) was determined using Prism software dose-response EC_{50} non-linear fitting by comparing drug-treated and untreated wells.

Standard Dose-response Inhibition Plaque Assays against Influenza H1N1

MDCK cells were pre-plated 24 hours in advance in 96-well plates. Serial dilutions of BSA-DA_{12} were prepared in DMEM with 2% FBS and 1% P/S and incubated with the influenza virus (Influenza A/Netherlands/2009 (H1N1), MOI=0.1) at 37 ºC for one hour, and then the mixtures were added to cells. Following the virus adsorption (1 h at 37 ºC), the virus inoculum was removed, the cells were washed, and the fresh medium was added. After 24 h of incubation at 37 ºC, the infection was analyzed with an immunocytochemical (ICC) assay. The cells were fixed and permeabilized with methanol. Then the Flu A monoclonal antibody (1:100 dilution) was added and incubated for 1 hour at 37 ºC. The cells were washed with wash buffer (PBS + Tween 0.05%) three times; then anti-mouse lgG, HRP-linked antibody (1:500 dilution) was added. After 1 hour, the cells were washed, and the 3,3'-Diaminobenzidine (DAB) solution was added. Infected cells were counted, and percentages of infection were calculated by comparing the number of infected cells in treated and untreated conditions. The concentration producing a 50% reduction in plaque formation (EC50) was determined using Prism software dose-response EC50 non-linear fitting by comparing drug-treated and untreated wells.

Standard Dose-response Inhibition Plaque Assays against SARS-CoV-2

The effect of ligand DA_{12} functionalized protein BSA against SARS-CoV-2 infection was evaluated by a dose-response plaque reduction assay. Vero E6 cells were plated in a 24-well plate around 24 hours in advance with a cell seeding density of 10^5 cells/well. Modified protein materials were prepared into 3 mg/mL concentration in miliq water. Six 1.5 mL Eppendorf tubes were prepared with 500 uL DMEM containing 2.5% FBS filled inside. Take 250 µL of 3 mg/mL sample and add into the first tube, mix well with 500 µL DMEM medium, then take 250 µL out from the first tube and transfer into the next one, 1 : 3 serial dilutions were performed like this until reaching the last tube. In the last tube after mixing, 250 µL volume was taken out and disposed of to keep every tube volume constant before incubation with viruses. Two hundred plaque-forming units (pfu) of SARS-CoV-2 viruses were added in each tube containing a different concentration of materials, mixed well, and incubated at 37 °C for 1 hour. Vero E6 cells culture medium was aspirated and added virus-material mixture after incubated for each well 200 µL and put it for incubation at 37 °C for 1 hour. Following virus adsorption, the virus inoculum was removed, and cells were washed with a medium, then overlaid with 500 µL of 0.4% Avicel GP3515 with DMEM 2.5% FBS. After incubation at 37 °C in the cell culture incubator for 48 hours, the medium was removed, and cells were fixed with 4% formaldehyde and stained with 500 µL 0.1 % of crystal violet in 20% ethanol for 20 mins, followed by washing with PBS 7.4 (1X) and drying the well. Viral plaques were counted manually. The concentration producing a 50% reduction in plaque formation (EC_{50}) was determined using Prism software dose-response EC_{50} non-linear fitting by comparing drug-treated and untreated wells.

Serum Influence on Dose-response Inhibition Plaque Assays against HSV-2

To evaluate the serum protein influence on antiviral materials to inhibit viral infection, 24.6 µL of these three types of protein-based antiviral materials BSA-DA_3, BSA-DA_6, and BSA-DA_{12} with a high concentration of 20.36 mg/mL were taken each and mixed with 30 µL of 100% FBS, making final serum protein percentage as 55%, the mixture of each sample was stirring with Thermomixer under 25 ºC at 600 rpm for 60 mins. Afterward, we took 27.3 µL of the mixture and diluted it with 723 µL of DMEM culture medium without FBS & P/S as the highest test concentration 333 µg/mL for antiviral. Then we perform a 1 : 3 serial dilution dose-response viral inhibition assay against HSV-2 as above. Viral plaques were counted. The concentration producing a 50% reduction in plaque formation (EC_{50}) was determined using Prism software dose-response EC_{50} non-linear fitting by comparing drug-treated and untreated wells.

Different antiviral treatment dose-response assay against HSV-2

Different antiviral treatment dose-response viral inhibition assays against HSV-2 were performed as follows:

(1) Pre-treatment: 250 µL of antiviral material BSA-DA_{12} with concentration 1 mg/mL was serial diluted by factor 1 : 3 with 500 µL of culture medium DMEM containing 2% FBS for 6 times, then HSV-2 viruses titer of 40000 pfu/mL was first diluted 5 times with DMEM medium with 2% FBS into 8000 pfu/mL, then took 10 µL adding into each tube contains different concentration of materials, mixed well, and incubated the mixture in the cell incubator under 37 ºC for 1 hour first, then the mixture was added into Vero cells for infection about 1 hour in the cell incubator under 37 ºC and 5% of CO_2. Then following with the standard virus's adsorption, washing, overlaying with methylcellulose, staining with crystal violet, and viral plaque counting as above.

(2) Co-treatment: 250 µL of antiviral material BSA-DA_{12} with concentration 1 mg/mL was serial diluted by factor 1 : 3 with 500 µL of culture medium DMEM containing 2% FBS for 6 times, then HSV-2 viruses titer of 40000 pfu/mL was first diluted 5 times with DMEM medium with 2% FBS into 8000 pfu/mL, then took 10 µL adding into each tube contains different concentration of materials, mixed well, and immediately directly added into Vero cells for infection about 1 hour in the cell incubator under 37 ºC and 5% of CO_2. Then following with the standard virus's adsorption, washing, overlaying with methylcellulose, staining with crystal violet, and viral plaque counting as above.

(3) Post-treatment: HSV-2 viruses' titer of 40000 pfu/mL was first diluted 5 times with DMEM medium with 2% FBS into 8000 pfu/mL, then took 10 µL adding into 500 µL of cell culture medium DMEM containing 2% of FBS. 200 µL of viruses containing medium was added into Vero cells for infection for 1 hour first in the cell incubator under 37 ºC. In parallel, antiviral material BSA-DA_{12} 625 µL of 1 mg/mL was added into 1250 µL of DMEM medium containing 1.2% methylcellulose for 1 : 3 serial dilution 6 times. Following virus adsorption, the virus inoculum was removed, and cells were washed with a medium and then overlaid with 500 µL medium containing 1.2% methylcellulose and different concentrations of antiviral material BSA-DA_{12} for incubation overnight in the cell incubator. Then next day followed with standard staining with crystal violet, and viral plaque counting as above.

(4) Cell-pretreatment: 250 µL of antiviral material BSA-DA_{12} with a concentration of 1 mg/mL was first serial diluted by factor 1 : 3 with 500 µL of culture medium DMEM containing 2% FBS 6 times, then we added 200 µL of DMEM medium containing different concentration of antiviral materials into Vero cells for incubation under 37 ºC with 5% CO_2 in the cell incubator for 1 hour first. At parallel, HSV-2 viruses' titer of 40000 pfu/mL was first diluted 5 times with DMEM medium with 2% FBS into 8000 pfu/mL, then took 10 µL adding into 500 µL of cell culture medium DMEM containing 2% of FBS. Following antiviral material cell penetration, Vero cells were washed by DMEM medium containing 2% FBS twice and replaced with 200 µL of DMEM medium containing viruses each well for infection 1 hour. Afterward, following with

the standard virus's adsorption, washing, overlaying with methylcellulose, staining with crystal violet, and viral plaque counting as above.

Flow cytometry assay for cell penetration

To evaluate antiviral material BSA-DA_{12} Vero cell penetration ability, we prepared native protein BSA and surface-modified protein BSA-DA_{12} with a concentration of 5.43 mg/mL. Fluorescent dye Alexa Fluor™ 647 NHS ester (10 mg/mL in anhydrous DMSO) 1.25 µL was added into protein solution 14.75 µL with equal stoichiometry and then shaken with an Eppendorf ThermoMixer at 25 ºC (600 rpm, 1 hour). The "labeled" mixture was used for the next step without purification. In this study, 10% of NH_2 in BSA protein was labeled. The dye-labeled mixture was further diluted 5 times into 1 mg/mL with PBS 1X. Vero cells were seeded with a density of 0.3 * 10^6 / well into 12 well plate one day in advance. Dye labeled BSA and BSA-DA_{12} with a final concentration of 1 mg/mL were added 10 ug each well into Vero cells, then incubated cell at 37 ºC and 4 ºC with 1 mL of DMEM medium containing 2% FBS for 1 hour (mimicking cell-pretreatment condition). Afterward, cells were washed with PBS 1X twice, harvested, and washed with FACS buffer (PBS 1X containing 0.2% BSA) 200 µL x 2. The cells were then stained with and resuspended in a DAPI solution (0.1 µg/mL, 200 µL) for flow cytometry analysis.

Confocal fluorescent microscope imaging for cell penetration

Native protein BSA and modified protein BSA-DA_{12} were prepared in PBS 1X solution with a concentration of 5.92 mg/mL. Fluorescent dye Alexa Fluor™ 647 NHS ester (10 mg/mL in anhydrous DMSO) 3.1 µL was added into protein solution 16.9 µL with equal stoichiometry and then shaken with an Eppendorf ThermoMixer at 25 ºC (600 rpm, 1 hour). The "labeled" mixture was used for the next step without purification. In this study, 20% of NH_2 in BSA protein was labeled. The dye-labeled mixture was further diluted 5 times into 1 mg/mL with PBS 1X. Vero cells were seeded with density 0.2 * 10^6 / well into 6 well plate one day in advance. A glass cover slide was put inside of the well during the seeding of the cells to let cells grow on top of it. Next day, dye-labeled BSA and BSA-DA_{12} with a final concentration of 1 mg/mL were added 20 µg each well into Vero cells, then incubated cell at 37 ºC and 4 ºC with 1 mL of DMEM medium containing 2% FBS for 1 hour (mimicking cell-pretreatment condition). Afterward, cells were washed by PBS 1X twice, and stained by Hoechst 33342 (10 µM) for nuclei staining in 1 mL of phenol/serum-free DMEM medium at 37 ºC for 15 mins followed by PBS washing (1 mL x 2). Vero cells were then fixed with 4% paraformaldehyde (PFA, 500 µL) for 15 mins at 37 ºC followed by PBS washing (1 mL x 2). Cell grown and stained cover slide was sealed onto a poly-lysine coated glass slid with 15 µL of ProLong™ diamond antifade mountant. The Vero cells were imaged with a Leica DMi8 with a 40X oil objective.

Evaluation of virucidal activity plaque assays against HSV-2

Vero cells were plated around 24 hours in advance with a seeding density of $2*10^4$/well in a 96-well plate. HSV-2 virus with titer 10^6 pfu/mL was used in this assay. Protein-based antivirals were prepared with a concentration of 300 μg/mL in 100 μL DMEM medium containing 2% FBS. 20 μL of titer 10^6 pfu/mL HSV-2 viruses were added into 100 μL materials, mixed well, and incubated at 37 °C for 1 hour. 96-well plate cell medium was first replaced with 100 μL of DMEM culture medium containing 2% FBS in each well. Three 1.5 mL Eppendorf tubes were first filled with 450 μL of DMEM medium containing 2% FBS, took 50 μL of virus-material mixture after incubation and added into the first tube, mixed well, then took another 50 μL from the first tube and transferred into the second one to make 1 : 10 serial dilutions, then 50 μL from the second one to the third tube. Three dilutions were prepared as 1 : 10, 1 : 100, and 1 : 1000. Afterwards, we took 50 μL of 1 : 10 dilution virus-material mixture and added into first-row number 1 and 2 wells, another 50 μL of 1 : 100 dilution into first-row number 3 and 4, third 50 μL of 1 : 1000 dilution into number 5 and 6, after that, we used multichannel pipette taking 50 μL from the first row and adding into second one, mix well, then 50 μL from second to third one until the seventh one for 1 : 3 serial dilutions. In the last row, add the virus-material mixture before any dilution of 50 μL into the first well, then 1 : 3 serial dilution until well number 6. After all these serial dilutions, we incubated the whole plate at 37 °C for 1 hour. Following virus adsorption, the virus inoculum was removed, and cells were washed with DMEM medium then overlaid with 100 μL medium containing 1.2 % methylcellulose. After incubation at 37 °C in the cell culture incubator overnight, the methylcellulose-containing medium was removed and cells were fixed and stained with 50 μL 0.1 % of crystal violet in 20% ethanol for 15 mins, followed by washing with PBS 7.4 (1X) twice and dry the well. Viral plaques were counted. Virus titers were calculated with dilutions at which the materials were not effective.

Evaluation of virucidal activity plaque assays against Influenza H1N1

MDCK cells were pre-plated 24 hours in advance in 96-well plates with a seeding density of $2*10^4$/well. Influenza virus (Influenza A/Netherlands/2009 (H1N1) (1.3 x 10^5 pfu/mL) and antiviral material BSA-DA_{12} with concentration 400 μg/mL were mixed in DMEM medium containing 2% FBS and 1% P/S and were incubated for 3 hours at 37 ºC. Serial dilutions of the virus-material complex together with the non-treated control were conducted and transferred onto the pre-seeded MDCK cells as above described. After 1 hour, the mixture was removed and the fresh DMEM medium with 1% P/S was added. The next day, viral titers were evaluated with ICC assay as described above.

Evaluation of virucidal activity plaque assays against SARS-CoV-2

Vero E6 cells were pre-plated 24 hours in advance in 24-well plates with a seeding density of 10^5/well. SARS-CoV-2 B.1.1.7 viruses with a titer of 10^5 pfu were incubated with antiviral material BSA-DA_{12} with a concentration of 1 mg/mL for 1 hour at 37 ºC. Serial dilutions of the virus-material complex together with the non-treated control were conducted and transferred onto pre-seeded Vero E6 cells as above described. After 1 hour, followed by virus adsorption, the virus inoculum was removed, and cells were washed with a medium, then overlaid with 500 µL of 0.4% Avicel GP3515 with DMEM 2.5% FBS. After incubation at 37 °C in the cell culture incubator for 48 hours, the medium was removed, and cells were fixed with 4% formaldehyde and stained with 500 µL 0.1 % of crystal violet in 20% ethanol for 20 mins, followed by washing with PBS 7.4 (1X) and drying the well. Viral plaques were counted. Titer was determined by counting plaques at the dilution for which BSA-DA_{12} material was not active.

MTS Cytotoxicity Assay

Protein-based antivirals' cell cytotoxicity was evaluated on Vero cells, MDCK cells, and Vero-E6 cells with CellTiter 96® AQ_{ueous} One Solution Cell Proliferation Assay (MTS). Cells were plated around 24 hours in advance in a 96-well plate with seeding density $2*10^4$/well in DMEM medium containing 10% FBS. Materials were 1 : 3 serial diluted 6 times with DMEM medium containing 2% FBS according to dose-response antiviral assay dilution factor, each diluted sample volume was kept into 200 µL. Replace the original cell culture medium with material containing a medium 200 µL each well and incubate at 37 °C in the cell culture incubator for 24 hours. After that, the material containing medium was removed and cells were washed with PBS 7.4 (1X) twice, then adding MTS reagents 10 µL + 90 µL DMEM serum-free medium into each well, incubate them at 37 °C for 4 hours. After incubation, absorbance at 490 nm was measured with a microplate reader Tecan. Cell viability ratio was calculated compared to non-drug-treated cells.

Statistical analysis

Statistical analysis was performed using GraphPad Prism 9 (GraphPad Software, Inc., La Jolla, CA, USA). Unless otherwise noted, the data are presented as Mean ± SEM. Comparisons of the two groups were performed by using a two-tailed unpaired Student’s t-test. Comparisons of multiple groups at a single time point were performed by using a one-way analysis of variance (ANOVA). P values were presented as $*P < 0.05$; $** P < 0.01$; $*** P < 0.001$; $**** P < 0.0001$.

Safety consideration

All the HSV-2 virus-related and Influenza H1N1 virus-related assays were performed in the biological safety level (BSL)-2 lab. All the SARS-CoV-2 virus-related assays were performed in the biological safety level (BSL)-3 lab. Personal protective equipment (PPE) is worn, and all procedures that can cause infection from aerosols or splashes are performed within a biological safety cabinet (BSC). An autoclave or an alternative method of decontamination is available for proper disposal. No unexpected or unusually high safety hazards were encountered.

Supplemental Figures

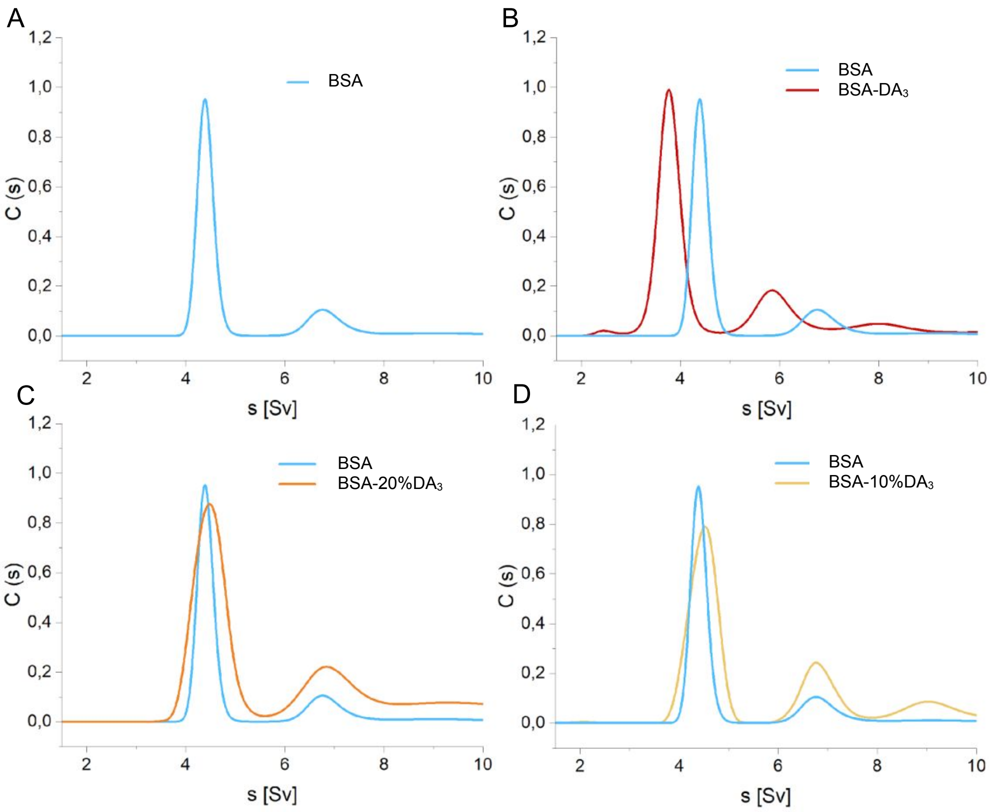

Figure S1. AUC analysis of different ligand density BSA-DA_3 samples. AUC sedimentation curve of BSA native protein (A); BSA-DA_3 with 56 ligands conjugated (B); BSA-20%DA_3 with 22 ligands conjugated (C); BSA-10%DA_3 with 13 ligands conjugated (D).

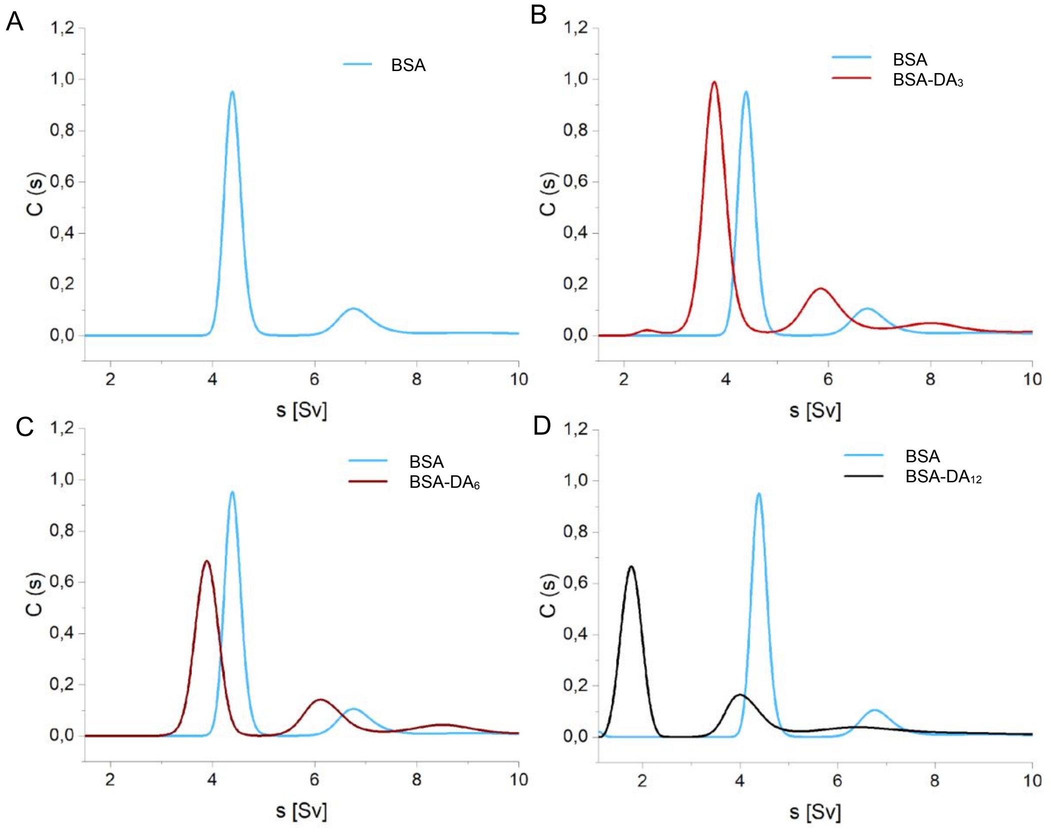

Figure S2. AUC analysis of different lengths of ligand DA_3, DA_6, and DA_{12} conjugated BSA protein. AUC sedimentation curve of (A) BSA native protein; (B) BSA-DA_3 with 56 ligands conjugated; (C) BSA-DA_6 with 37 ligands conjugated; (D) BSA-DA_{12} with 22 ligands conjugated.

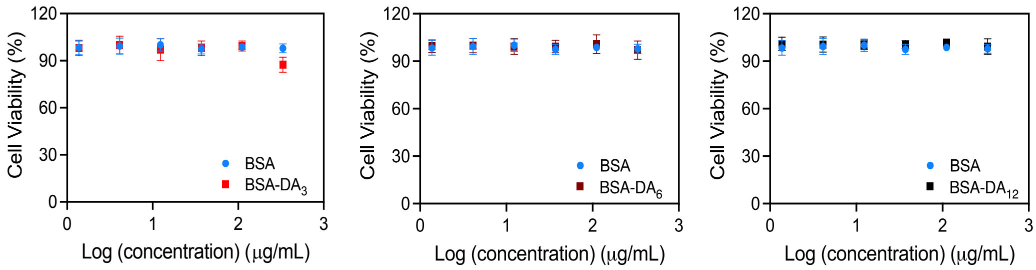

Figure S3. Cell cytotoxicity of different lengths of ligand DA_3, DA_6, and DA_{12} conjugated BSA protein antivirals on Vero cells. Three different ligands conjugated BSA proteins together with its native version were tested for cytotoxicity using the standard MTS Cytotoxicity Assay. Statistical analysis was performed and is presented as Mean ± SEM.

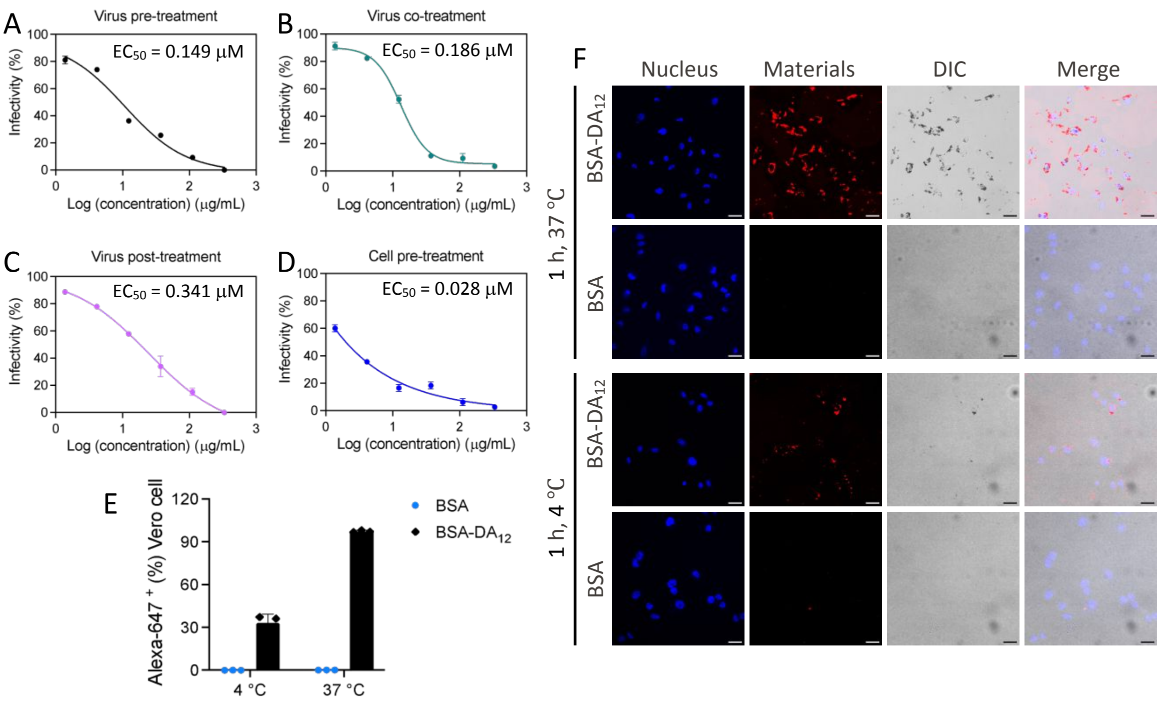

Figure S4. Different viral inhibition treatments of BSA-DA$_{12}$ influence on antiviral inhibition effect against HSV-2. Does-response viral infectivity of HSV-2, (A) viruses were pre-treated by BSA-DA$_{12}$ for 1 hour before infection; (B) viruses were co-treated with BSA-DA$_{12}$ by adding together for infection immediately; (C) viruses were adding to host cells for infection for 1 hour before adding BSA-DA$_{12}$; (D) BSA-DA$_{12}$ were adding to host cells 1 hour before viruses were added for infection; (E) BSA-DA$_{12}$ Vero cell penetration at both 37 °C and 4 °C by flow cytometry; (F) Confocal imaging visualization of BSA-DA$_{12}$ Vero cell penetration at both 37 °C and 4 °C, Scale bar: 30 μm, the images were obtained via Leica DMi8 with a 40X oil objective.

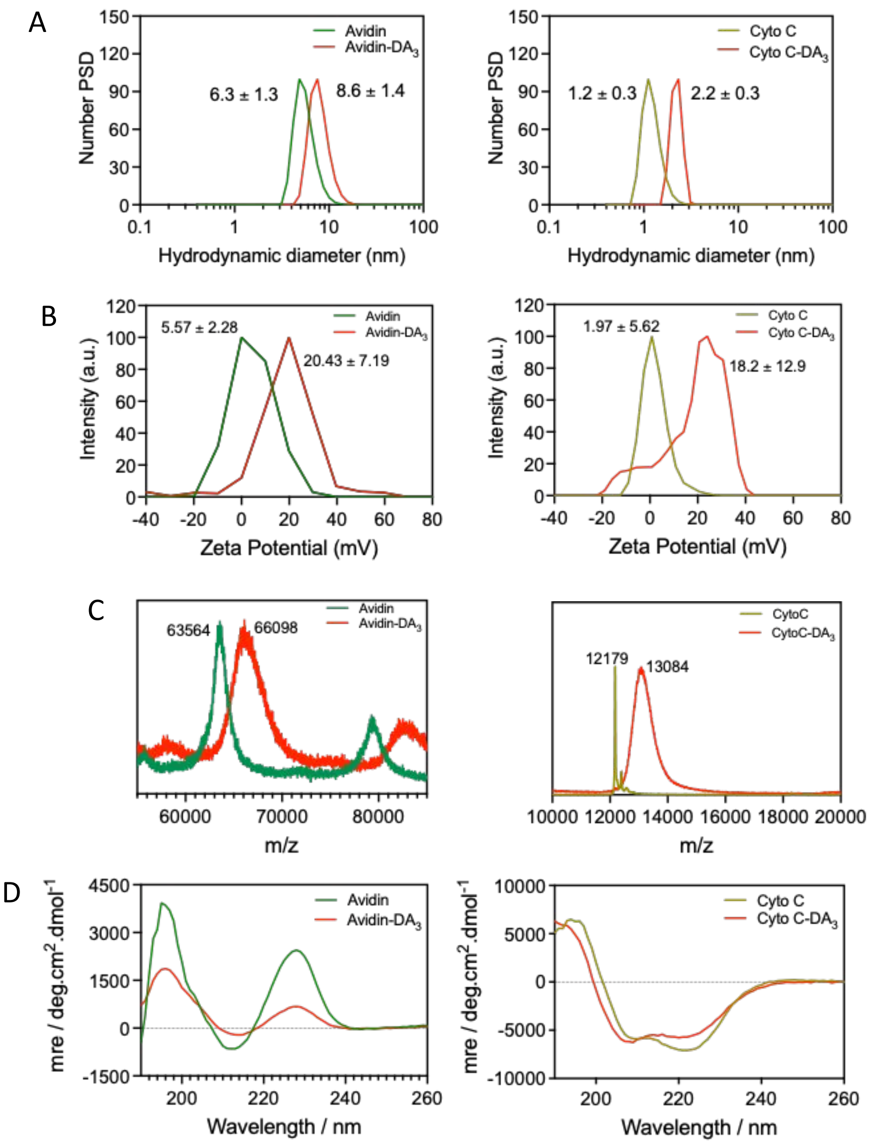

Figure S5. Characterizations of DA_3 modified Avidin and Cyto C. (A) Size distribution measurement of modified Avidin and Cyto C by DLS. (B) Surface zeta potential of Avidin, Cyto C and Avidin-DA_3, Cyto C-DA_3. (C) MALDI-TOF mass spectra overlay of native and modified protein of Avidin and Cyto C. (D) CD spectra of native and modified protein structure of Avidin and Cyto C.

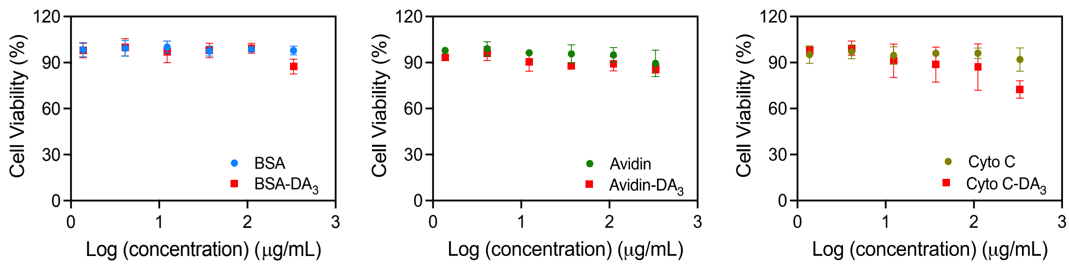

Figure S6. Cell cytotoxicity of different protein core-based antivirals on Vero cells. Three proteins, BSA, Avidin, and Cytochrome C, along with their chemically modified versions, were tested for cytotoxicity using the standard MTS Cytotoxicity Assay. Statistical analysis was performed and is presented as Mean ± SEM.

(1) Mathez, G.; Cagno, V. Clinical Severe Acute Respiratory Syndrome Coronavirus 2 Isolation and Antiviral Testing. *Antivir. Chem. Chemother.* **2021**, *29*, 1–9. https://doi.org/10.1177/20402066211061063.
